# P-1854. Implementation of an Inpatient Service for Facilitation of Transition of Care for Outpatient Antimicrobial Therapy (OPAT) Patients

**DOI:** 10.1093/ofid/ofaf695.2023

**Published:** 2026-01-11

**Authors:** Anila Hussain, Lisa Pedroza, Madeline King, Kelly E Rowe, Dana D Byrne, Sum Chi Lydia Poon

**Affiliations:** Rhode Island Hospital, East Greenwich, RI; Cooper Medical School Rowan University, Camden, New Jersey; Cooper University Hospital, Camden, New Jersey; Cooper Medical School of Rowan University, Camden, New Jersey; Cooper University Hospital, Camden, New Jersey; Cooper Medical School of Rowan University, Camden, New Jersey

## Abstract

**Background:**

Outpatient Parenteral Antimicrobial Therapy (OPAT) allows patients to complete extended courses of IV antibiotics in an outpatient setting. We established a formal inpatient OPAT transition service led by an infectious disease (ID) physician and an ID pharmacist. The goal was to improve transitions of care from inpatient to outpatient setting and assess barriers to discharge, including antimicrobial selection and insurance coverage.

**Figure:**
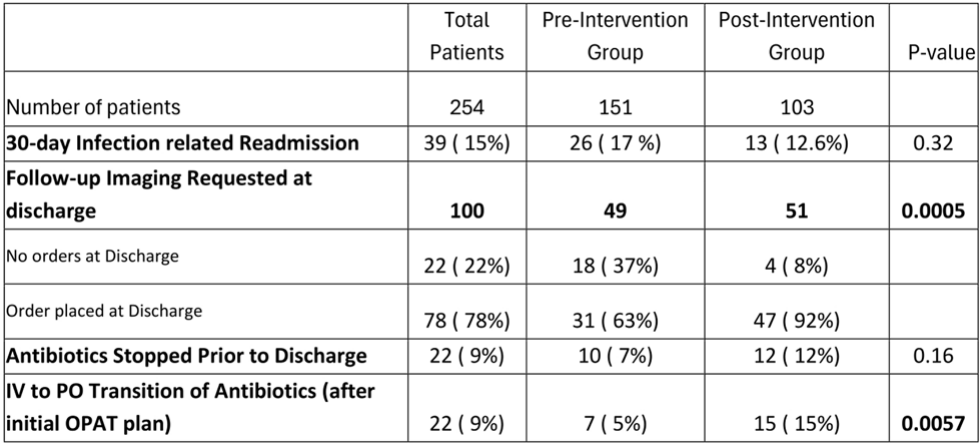
OPAT Pre and Post Intervention Outcomes Table

**Figure:**
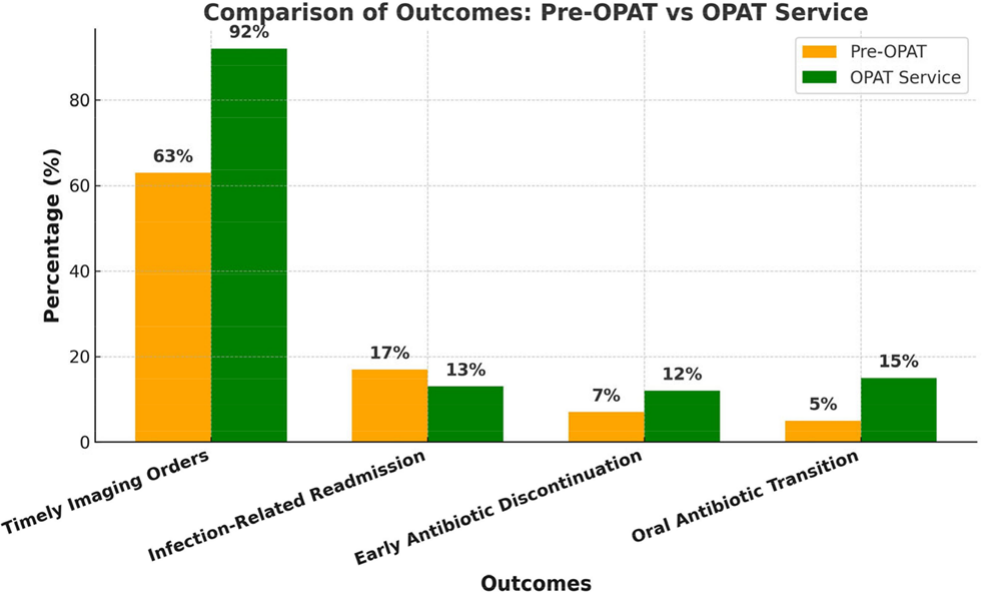
OPAT outcomes Graph

**Methods:**

We conducted a retrospective pre-intervention (May – July) and post-intervention (August – October) chart review to assess the effects of the program. We compared following outcomes between both groups; medication changes due to side effects or updated clinical data, ordering and completion of recommended follow-up imaging in the requested timeline, transition to oral antibiotics or cessation of antibiotics based on clinical response, and 30-day readmission data related to infection. T-test and Chi-square tests were used to compare variables

**Results:**

A total of 254 patients were included in the analysis. The average age of patients was 57.7 years and the most common diagnosis in both groups was bacteremia. Antibiotic adjustment based on new culture data or imaging results showed no difference between the 2 cohorts. Timely imaging orders at discharge improved from 63% (31/49) to 92% (47/51) in the OPAT service group (p = 0.0005) and infection-related readmission decreased from 17% (26/151) to 13% (13/103) (p=0.32). The OPAT service appropriately stopped antibiotic therapy in 12% of patients compared to 7% being stopped in the pre-intervention group (p=0.16). In the post-intervention group, 15% of patients were transitioned to oral antibiotics compared to 5% in the pre-intervention group (p=0.0005)

**Conclusion:**

OPAT transition service initiation led to timely imaging orders, a decrease in infection-related readmissions, appropriate antibiotic discontinuation, and transition to oral antibiotic regimen. We regularly assess our OPAT transition service to improve its efficacy and efficiency, and to ensure optimal patient safety. Follow-up data is currently being collected to assess further effects and long-term impact of our OPAT transition service

**Disclosures:**

Madeline King, PharmD, Innoviva: Advisor/Consultant|Shionogi: Advisor/Consultant Dana D. Byrne, MD, MSc, Merck: I am currently employed by Merck but this research was conducted prior to my employment|Merck: Stocks/Bonds (Private Company)

